# Supplementary material for: Validation study of HLA-B*13:01 as a biomarker of dapsone hypersensitivity syndrome in leprosy patients in Indonesia
Source: PLoS Negl Trop Dis. 2020 Oct 16;14(10):e0008746. doi: 10.1371/journal.pntd.0008746 (PMC7592909; doi:10.1371/journal.pntd.0008746)
Supplement: Attachment — Submitted filename: 20200818 Rebuttal Final.docx [file pntd.0008746.s002.docx]

**Rebuttal for Submission No**: PNTD-D-20-00734

**Manuscript Title:** Validation study of HLA-B*13:01 as a biomarker of dapsone hypersensitivity syndrome in leprosy patients in Indonesia

We thank all reviewers for providing their insights and suggestions to improve our manuscript.

Before responding to each reviewer’s comments, we would like to inform you of the change in some statistical results. While re-analyzing the study results to include covariates as suggested by Reviewer #1, we realized a mistake in quoting the statistical P-value and odds ratio of the *HLA-B*13:01*’s association to DHS due to a faulty statistical correction in the bioinformatics script. We regret and deeply apologize for this mistake and had made the proper correction in this revised manuscript. Summary of our changes are underlined as follows:

1. In the Abstract section

changed “…*odds ratio=247.6 and P-value=4.81×10^-9^*…”

into

“…*odds ratio=233.64 and P-value=7.11×10^-9^*…”

2. In the Results section

changed “*(odds ratio=247.6 and P-value=4.81×10^-9^)*”

into

“*(odds ratio=233.64 and P-value=7.11×10^-9^)*”

3. In the Results section

Revised P-values and odds ratios of the 30 HLA-B alleles in Table 2. Please see revised Table 2 for the changes.

4. In the Discussion section

changed “*…the odds ratio we discovered in Papuans are 12-times what was reported in Chinese and 4.6-times what was reported in Thais*”

into

“*…the odds ratio we discovered in Papuans are 11-times what was reported in Chinese and 4.3-times what was reported in Thais*.”

Please find below a point-by-point response to each of the comments in blue.

Editor

While revising your submission, please upload your figure files to the Preflight Analysis and Conversion Engine (PACE) digital diagnostic tool, https://pacev2.apexcovantage.com. PACE helps ensure that figures meet PLOS requirements.

Response: We have replaced Fig 1 and 2 in the tracked- and clean-version of the manuscript with a better resolution and the figure has passed PACE digital diagnostic tool as seen from the screenshot below. Fig 1 on top, followed by Fig 2.

Reviewer #1:

Major and general comments: This is an important area and there are potentially interesting findings here in order to draw appropriate conclusions from the data the analysis should be redone and that much more of the actual data including the causality assessment and functional data should be included for the readers (supplement).

Response: Thank you for your comments.

1. In terms of the analysis at a minimum this should include matching cases to age, sex, race and match for underlying disease state and co-morbidities and conditional logistical analyses. functional data and matched analysis would be enormously helpful to clean this up so conclusions can be drawn from the data.

Response: Thank you for your comments. Although we did not purposely match every single case to a control in our study, the difference in the distribution of age, sex and region were not statistically significant in our case and control groups (see new Table 1). We had also mentioned in the Methods section that “*Each case has at least one control obtained from the same area.*”

To strengthen the argument and evidence to support your suggestion, we inserted the following information into the revised manuscript:

1. In Methods, we added
   a. “*Information of age, gender and province were collected from each subject.*” under Study Population section.
   b. *Subjects who have any comorbidities or are under other types of medication other than the standard MDT were excluded from the study.*” under Subject Recruitment section.
   c. “*The statistical difference between the age distribution in cases and controls was calculated based on Student's t-test, while gender and geographical distribution was calculated based on Pearson’s chi-square test...*” under Statistical Analysis section.
2. In Results, we added
   a. “*As study subjects were recruited with a uniform criteria and matched according to the geographic region,* *the difference between the distribution of gender and age in cases and controls were not statistically significant (P>0.05, Table 1). This minimizes the chances of the genetic association results being attributable to artefacts from baseline characteristics of the study subjects*.” in the first paragraph.
   b. Added a new table: “*Table 1. Baseline characteristics of cases and controls*”

c. Added columns for OR and P-values after adjusting for covariates (age, sex, region)

2. With regards to previous study showing an association between HLA-B*13:01 has been described in association with co-trimoxazole-induced DRESS (PMID: 32452529), do you have cases or control using co-trimoxazole?  It should be discussed cross reactivity between Dapsone and co-trimoxazole.

Response: In this study, patients with co-morbidities is one of the exclusion criteria of the subject recruitment. We made this clearer in the revised manuscript by adding the following sentence in the ‘Subject Recruitment Criteria’ section under Methods: “*Subjects who have any comorbidities or are under other types of medication other than the standard MDT were excluded from the study.*”

3. The previous study showed that HLA-B*13:01 was the predictive markers for both DRESS and SJS-TEN. In this study the authors should reanalyze to confirm the phenomenon.

Response: In this study, we followed previously published guideline in 1989 on how to diagnose dapsone hypersensitivity: *Richardus JH, Smith TC. Increased incidence in leprosy of hypersensitivity reactions to dapsone after introduction of multidrug therapy. Lepr Rev. 1989;60(4):267-73*. Likewise, the first publication about the association of *HLA-B*13:01* and DHS in New England Journal of Medicine also followed the same criteria, which includes the presence of skin eruptions, fever, lymphadenopathy and hepatic involvement. Symptoms must also disappear upon the discontinuation of dapsone. With this checklist we categorized leprosy patients as having DHS and included them in the study.

Unfortunately, we did not denote percentage of mucosal involvement and the presence of flat, diffuse erythematous maculopapular rashes that distinguishes DRESS against SJS-TEN. Thus, we are unable to perform the stratified analysis as you suggested.

4. As too many HLA-B genotypes were discovered and compared, the p-value should be corrected by Bonferroni correction.

Response: There are 30 HLA variants that were discovered and compared. By performing a Bonferroni correction, this would bring the P-value significance threshold down to P<0.0016 (i.e. 0.05/30). In our study, the P-value of *HLA-B*13:01*’s association to DHS is 7.11×10^-9^. Applying Bonferroni correction, it becomes P=2.13×10^-7^, which is smaller than the Bonferroni corrected significance threshold. Thus, the association of *HLA-B*13:01* to DHS remains to be significant.

We also made these revisions to support your suggestion:

a. In Methods – Statistical Analysis, we added: “*Statistical significance is defined as P<0.0016 after considering Bonferroni correction for multiple testing of 30 HLA-B alleles.*”
b. Footnotes under Table 2, we added: “*Considering Bonferroni correction for testing of 30 HLA-B alleles, the P-value for significance is P<0.0016.”*

5. Table 1 showed and compared alleles frequency of HLA-B genotype. As we known that carry only one allele can be the risk individual, the number of carrier should be expressed and analyzed.

Response: Thank you for your comments. We have described the carrier vs non-carrier counts in the previous Table 2 (now Table 3 due to the addition of Table 1 for baseline characteristics). We apologize if the Table was previously not very clear. Therefore, we have revised the table to include the word ‘Carriers’ above Heterozygous and Homozygous genotype carriers and added a new column “Total carriers” to sum the total of carriers in cases (‘DHS+’) and controls (‘DHS-‘) groups.

Under the same section we’ve also included the risk analysis of carriers vs non-carriers. Please refer to this sentence “*Consequently, the risk carried by a person who has at least one copy of HLA-B*13:01 allele (heterozygous odds ratio) is then 233.3 times higher than the risk of those who do not carry any copy of HLA-B*13:01 allele.”*

6. The general population from the same race should be recruited and genotyped to compare and it can help to extrapolate for screening in clinical implementations.

Response: Thanks for your comments. There are a few considerations why we thin *HLA-B*13:01* is only meant for screening in leprosy population rather than the general population:

(1) dapsone is only most often used to treat leprosy, and rarely found to treat other diseases in Indonesia;
(2) the leprosy patient population in Indonesia is small (~20,000 people);
(3) the leprosy patient population are clustered and could be easily identified for screening;
(4) cost-effectiveness may only be achieved by screening a target population rather than the whole population;
(5) like other pharmacogenomic markers such as *HLA-B*15:02* for carbamazepine-induced SJS/TEN, governments around the world generally reimburses only patients who are about to be administered the drug (i.e. reactive screening instead of pre-emptive), likewise we see *HLA-B*13:01*’s screening being most beneficial to be implemented as a reactive testing.

With the above considerations, we strongly believe that *HLA-B*13:01* screening for DHS will be most beneficial to be screened in a targeted population, i.e. leprosy patients, instead of the general population.

Nevertheless, we have indicated in the manuscript some data about the general population’s *HLA-B*13:01* allele frequency as this information gives the readers some sense of the amount of risk present in the average population. We mentioned in the last sentence of the first paragraph of Discussion: “*In Chinese, the frequency of HLA-B*13:01 ranges between 3-8% across North and South China, but for the Melanesians and Australian Aboriginals who are evolutionary related to Papuans, the allele frequency may reach up to 28%.*”

Below we also provide more evidence and comparison between the frequency of *HLA-B*13:01* in our study population and a curated public database, for your reference.

7. HWE was less than 0.05, the authors should describe and discuss.

Response: Thanks for your comments. We have described a sentence about HWE in the first paragraph of the Results section: “*However, we also observed a significant deviation of the genotype frequency of HLA-B*13:01 in the case patients (P=0.00039), unlike the controls (P=1).*” and furthermore expanded our interpretation of these results in the third paragraph of the Discussion section: “*Similar to the publication in the Chinese population, we found a significant deviation of HLA-B*13:01 genotype frequency from Hardy Weinberg equilibrium in only the case patients (P=3.9×10^-4^). We also think the reason for this is due to the potential oversampling as the effect of the highly penetrant HLA-B*13:01 as a susceptibility factor for DHS and the bias towards sampling only the surviving DHS patients. Although not very well documented, the mortality rate for DHS in Papua is estimated to be quite high. Many of the patients come from rural areas and oftentimes they were treated after active case findings into their home villages. Upon drug delivery, any side effect that occur to them were often not reported. Patients even think these side effects were curses instead of a medical condition. Hence, with not much understanding of the disease and potential side effects, many of the fatal cases would have been missed and being under-reported.*”

8. The clinical characteristics for cases-controls should be draw out. Recently, the underlying condition such as renal function can determine the risk of drug-induced SCARs such as allopurinol. Dose can also the risk factors. The authors should reanalyze the non-genetics risk factors.

Response: Dapsone hypersensitivity syndrome has been categorized as a dose-independent (idiosyncratic) adverse effect by multiple literature (Lepr Rev. 2001 Mar;72(1):57-62.; Clin Exp Dermatol. 2003 Sep;28(5):496-8; Clin Rev Allergy Immunol. 2003 Jun;24(3):229-38; J Pharmacol Pharmacother. 2013 Apr-Jun; 4(2): 158–160.; Am J Trop Med Hyg. 2017 May 3; 96(5): 1014–1018.). Besides, dapsone is also given at a standard dose to all leprosy patients following WHO’s standard guidelines for administering leprosy’s MDT blister pack. Thus, in terms of dose, it is unlikely to be a risk factor for DHS.

As for other underlying functions, to date, we have only found two case studies reporting the suspicion of hepatitis B (Int J Lepr Other Mycobact Dis. 1999 Jun;67(2):171-2.) and hepatitis E (Indian J Gastroenterol. Apr-Jun 2000;19(2):85-6.) as risk factors for DHS. Hepatitis as a risk factor for DHS was further reported in a systematic review (Acta Derm Venereol. 2012 Mar;92(2):194-9), but the association P-value of hepatitis and DHS was not significant after adjusting for age and gender (P=0.07). Since we have excluded any patients with co-morbidities in our study, this underlying condition is no longer a modifying factor.

9. The authors should show number need to test to determine how many patients would be needed to screen to identify one patient likely to go onto DHS.

Response: Thanks for your comments. We have this information on the last sentence of results section “*HLA-B*13:01* as a risk predictor for DHS in Papuans”, saying “*This translates to a PPV of 74.8%, NPV of 98.9%, and 10 people needed to screen to prevent one case of DHS.*”

10. Please check through the manuscript for genetic format, it should be italic form.

Response: Thanks for your detailed observation. We have reformatted all mentions of HLA-B gene and HLA-B alleles into italic form throughout the manuscript.

11.The first paragraph of Introduction (line 62-65), second paragraph (line 75-78) need reference.

Response:

Line 62-63: *“Multi-drug treatment (MDT) supplied by the World Health Organization is intensively applied as the main treatment of leprosy in every region in Indonesia.”*

- Added reference: ‘Global leprosy update, 2016: accelerating reduction of disease burden. Wkly Epidemiol Rec. 2017;92(35):501-19.’

Line 64-65: *“Although the availability of data is scarce, many cases of Dapsone Hypersensitivity Syndrome (DHS) were reported from districts in Papua and oftentimes are fatal cases.”*

- Added references:

‘Pongtiku A, editor Evaluasi Annual Progress Report 2015 Papua dan Papua Barat. PL Meeting; 2016; Bali, Indonesia.’

and

‘Yokhu YB, Giyai A. Laporan Tahunan 2014 Program Pengendalian Kusta Provinsi Papua. Jayapura, Papua, Indonesia: Dinas Kesehatan Provinsi Papua; 2014.’

Line 75-78: “*The syndrome is a severe idiosyncratic drug reaction characterized by the clinical manifestations of fever, rash, and systemic involvement (most commonly of the liver and the hematologic system), which can cause severe organ dysfunction. DHS is usually manifested 4 to 6 weeks after the initiation of therapy.”*

- Added reference: ‘Richardus JH, Smith TC. Increased incidence in leprosy of hypersensitivity reactions to dapsone after introduction of multidrug therapy. Lepr Rev. 1989;60(4):267-73.’

12. As the authors mentioned that the leprosy patients got multi-drug treatment, how can the culprit Dapsone as drug-induced hypersensitivity, the Elispot and LTT should be performed to confirm the DHS.

Response: In our study, we used the criteria proposed by Richardus and Smith (*Richardus JH, Smith TC. Increased incidence in leprosy of hypersensitivity reactions to dapsone after introduction of multidrug therapy. Lepr Rev. 1989;60(4):267-73.*) to diagnose DHS. One of the criteria reads “*the symptoms appeared within 8 weeks after commencement of dapsone and disappeared after discontinuation of the drug*” – line 130 of the newly revised manuscript. Although we were not able to perform Elispot and LTT, in our study, all of our cases have had their symptoms appearing within 8 weeks and dapsone was withdrawn from their treatment regime where symptoms started to improve -line 131 of the newly revised manuscript. As we recruited retrospective case subjects, during the point of recruitment all of the subjects have resumed and completed their MDT course with dapsone being cut-out from their blister packs. They did not exhibit any adverse symptoms during this 2-drug course. As for the control subjects, they have completed the standard MDT course of 3-drugs without having any adverse reactions. –refer to Study Design section under Methods.

13. What is the criteria to define DHS.

Response: The following criteria proposed by Richardus and Smith (*Richardus JH, Smith TC. Increased incidence in leprosy of hypersensitivity reactions to dapsone after introduction of multidrug therapy. Lepr Rev. 1989;60(4):267-73*.) were used in our study for the diagnosis of DHS: (1) showing at least two of the following symptoms: 1. fever, 2. eruptions, 3. lymphopathy, 4. hepatic function abnormalities (hepatomegaly, jaundice and/or hepatic dysfunction); (2) symptoms and signs appearing linked with the administration of dapsone, and clinical improvement along with the withdrawal of the drug; (3) symptoms not attributed to other drugs, or leprosy reactions; (4) excluding other diseases causing similar manifestations.

14. The causality assessment should be performed and express in table format.

Response: To date we have not seen any literature recommending a causality assessment in determining the diagnosis of DHS, neither is it being used in actual clinical management of leprosy patients. Nevertheless, since the most important information from a causality assessment is the certainty whether the drug is the causal drug for the adverse symptoms, during subject recruitment and case/control classification, we followed closely the guidelines by Richardus and Smith (1989) mentioned above, which briefly includes the presence of skin eruptions, fever, lymphadenopathy and hepatic involvement. Symptoms must also disappear upon the discontinuation of dapsone. With this checklist we categorized leprosy patients as having DHS and included them in the study.

What is certain in our study recruitment as also mentioned above is that the retrospective case subjects exhibited DHS symptoms within 8-weeks of the 3-drug MDT treatment, but at the point of recruitment, they had resumed and completed their 2-drug MDT course with dapsone being cut-out from their blister packs while they did not exhibit any adverse symptoms. As for the control subjects, they have completed the standard MDT course of 3-drugs without having any adverse reactions. Thus, we are certain that dapsone was the causal drug in the hypersensitivity syndrome that was being studied in this paper.

Reviewer #2:

1. The paper “Validation study of HLA-B*13:01 as a biomarker of dapsone hypersensitivity syndrome in leprosy patients in Indonesia” Krismawat et al evaluates the allele HLA-B*13:01 association with dapsone hypersensitivity among Indonesians. This data is not original but an important replication of previously described among Chinese Thai and other populations. The paper is well written and aims are correctly addressed. The sample size is small but due to the high odds ratios expected, calculations supported the numbers with good power estimations. The genotyping methods for HLA results were performed adequately and logistic regression is the best analysis approach to test association.

Response: Thank you for your comments.

2. The findings are important and deserve publication. The results are very clear and provides good evidence to screen the Indonesian population prior to dapsone treatment avoiding life-threatening conditions for leprosy patients.

Response: Thank you for your comments.

3. Authors must discuss a little better the introduction of this screening as a policy and a great improvement would be cost-effectiveness analysis to evaluate impact in incidence and mortality. Indeed, a recent publication listed by authors performed a good prospective study and authors cited as “Screening for this allele in new leprosy patients before the initiation of MDT has proven beneficial to reduce incidence rate of DHS in China to zero”. Authors should consider follow up and complementary to this previous analysis, with cost-effectiveness analysis.

Response: Thank you for your comments. We added a few sentences in the last paragraph of the Discussion section as follows “*We believe that by implementation of a cost-effective genomic test, health programs can prevent DHS occurrence to close to zero, thereby increasing MDT’s safety and confidence as a standard of care, while demonstrating cost savings to the health care system and relieving an enormous burden on quality of life and potential death on patients and their families.*”.
